# Effect of Coriander Plants on Human Emotions, Brain Electrophysiology, and Salivary Secretion

**DOI:** 10.3390/biology10121283

**Published:** 2021-12-06

**Authors:** Wenzhu Zhang, Zhaoming Li, Lingshan Wang, Hui Liu, Hong Liu

**Affiliations:** 1 Beijing Advanced Innovation Center for Biomedical Engineering, Institute of Environmental Biology and Life Support Technology, School of Biological Science and Medical Engineering, Beihang University, Beijing 100083, China; zhangwenzhu29@163.com (W.Z.); lizhaoming@buaa.edu.cn (Z.L.); 16231027@buaa.edu.cn (L.W.); lh64@buaa.edu.cn (H.L.); 2 International Joint Research Center of Aerospace Biotechnology & Medical Engineering, Beihang University, Beijing 100191, China

**Keywords:** coriander plant, emotion, brain electrophysiology, salivary secretion

## Abstract

**Simple Summary:**

This research aims to investigate the effects of coriander plants on human emotions and physiological activities. The results showed coriander plants could significantly reduce the angry sub-scores, alpha amylase and amino acids (arginine, proline, histidine, and taurine) concentrations in saliva. Theta (4–8 Hz) band activity of the cerebral cortex was significantly enhanced. Moreover, taurine significantly positively correlated with anger and negatively correlated with vigor. All the results signified that coriander plant could influence the activity of brain electrophysiological and salivary secretion through its VOCs to improve people’s negative emotions. This study will provide a theoretical basis for the living coriander plants have some therapeutic effect on the human psychological state.

**Abstract:**

Coriander is a popular herb with versatile applications. However, the current research about coriander medicinal values have been mainly focusing on its extracts while lacking in the relationship between living coriander plants and emotion. Therefore, this study aims to investigate the effects of coriander plants on human emotions and physiological activities. The results showed that the main Volatile organic compounds (VOCs) of coriander plants were 2-ethyl-1-hexanol, d-limonene, eucalyptol, benzyl alcohol, Isophorone, dimethyl glutarate, α-terpineol, styrene, methyl methacrylate, α-pinene. Coriander plants could significantly reduce the angry sub-scores, alpha amylase and amino acids (arginine, proline, histidine, and taurine) concentrations in saliva. Theta (4–8 Hz) band activity of the cerebral cortex was significantly enhanced. Moreover, taurine significantly positively correlated with anger and negatively correlated with vigor. All the results signified that coriander plant could influence the activity of brain electrophysiological and salivary secretion through its VOCs to improve people’s negative emotions.

## 1. Introduction

People in modern society are under greater life and work pressures, which will affect their physical and mental health and even increase the risk of depression [1,2]. Depression is a persistent and serious mental illness that affects over 120 million people worldwide [2]. According to the World Health Organization statistics, depression will be the leading cause of death worldwide by 2030 [3,4]. According to the reports, the direct or indirect treatment costs of depression are more than $30 billion each year in the United States, which will lead to a significant social burden [5]. Moreover, most of the treated depression patients often have residual symptoms that persist, leading to impaired social function of the patients. Therefore, the prevention of depression is particularly important [6].

In stressful modern life, the relaxing effect of natural stimuli is considered beneficial compared with other stimuli [1]. According to stress recovery theory, exposure to unthreatening nature can elicit positive emotions, restrict negative thoughts, and reduces stress [7]. Many people are thus attracted to the physiological and psychological relaxing effect of exposure to plants. Field experiments in urban parks and forest bathing have proved the psychological and physical relaxing effects of contact with plants [8,9]. Forest bathing also increased natural killer cell function and improved immune function [10]. This effect was sustained for approximately 1 month. Indeed, urban residents were socially isolated during the outbreak of the Covid-19 virus outbreak, and the value of residential gardens as therapeutic landscapes was brought to the fore [11]. These results suggest that contact with plants is a type of prophylaxis.

The color and volatile organic compounds (VOCs) of plants may play an important role in regulating emotions. People mainly achieve the color perception through light, and different colors of plants reflect different wavelengths of light [12]. Intrinsically photosensitive retinal ganglion cells (ipRGCs) sense light and project it to the paraventricular nucleus of the hypothalamus. This area regulates the secretion of stress hormones by stimulating the adrenal gland [13]. Stress hormone levels can reflect stressful, emotional conditions [14]. Previous research has shown that the green color of plants can effectively restore workers’ brainwave and the mental state, reduce shoulders and back pain, and relieve their work pressure [15]. The volatile organic compounds of plants are beneficial to people’s physiological and psychological health. They can relieve anxiety and depression and maintain the memory of patients with Alzheimer’s disease or other memory disorders [16].

Coriander or *Coriandrum sativum* (*C. sativum*), a member of the Apiaceae family, is a popular herb with versatile applications. The seeds and leaves are widely used for culinary and seasoning. The seeds and fruits are often used for cooking meat [17]. As honey plant, coriander is highly attractive to honeybee workers [18]. Additionally, coriander has been used in many traditional medicines, and its medicinal values has been widely recognized [19,20]. Coriander extracts have a wide range of biological benefits including neuroprotective, anxiolytic, hypnotic, antioxidant, anti-inflammatory, and so on [21,22]. However, the current research about coriander medicinal values have been mainly focusing on its extracts while lacking on living coriander plants. Our previous study found that coriander plants had a potential role in regulating negative emotions [12]. Therefore, this study aims to investigate the effects of coriander plants on human emotions and physiological activities and the correlation between emotional fluctuations and salivary secretion. This study will provide a theoretical basis for the living coriander plants have some therapeutic effect on the human psychological state.

## 2. Methods

### 2.1. Participants

Participants were nonsmokers with no history of physical or mental illness. Alcohol, tobacco, and caffeine intake were prohibited throughout the experimental period. 26 college students aged 24 ± 2 years (13 males and 13 females). Before the experiment began, the participants were informed of the experimental procedure, excluding the purpose of the study, and they signed informed consents. This study was approved by the Science and Ethics Committee of School of Biological Science and Medical Engineering, Beihang University, Beijing, China (Approval ID: BM20200058; Date: 6 January 2020).

G*Power software 3.1 was selected to calculate the sample size. The effect size of the calculation parameters indicated the influence of an independent variable on the result, generally obtained from experience or existing experiments. The Cohen’s d was 1.2, the α error probability was 0.05, power (1-β error probability) was 0.8. The sample size of this experiment was calculated to be 24. The actual number of participants tested matches the calculated sample number. The 26 participants were divided into control group and coriander group with the same number of male and female. There were no significant differences in age, BMI, and self-reported anxiety and depression scores between the two groups (Table 1).

### 2.2. Coriander Plant Material

Coriander (*Coriandrum sativum* L.) was an herb of the genus *Coriandru* of the Umbelliferae family. Large-leaved variety was selected to cultivation, which seeds were purchased from a commercial source in Beijing, China, and authenticated at the Institute of Environmental Biology and Life Support Technology, Beihang University. Coriander plants were grown in planting pot (43 cm × 19 cm) in an environmentally controlled chamber. Each planting pot contains 8 coriander plants. Using white planting pots to reduce the visual impact. The temperature was at constant 20 °C, the photoperiod was 16 h light, and relative humidity was around 55%. The coriander was transferred to the test room for VOCs collection when it was 40 days old. VOC was collected after 48 h of acclimatization cultivation. The collection time was 2:00 p.m. coinciding with the test time. No participants were tested at the time of VOCs collection.

### 2.3. Collection and Detection of VOCs from Coriander Plants

VOCs of coriander plants were collected using a QC-1B atmospheric sampler (Beijing Ke’an Labor Protective New Technology Company, Beijing, China). The atmospheric sampler was connected to a TVOC sorbent tube containing Tenax-TA sorbent. The gas circulation flow rate was 100 mL/min and the extraction duration was 1 h.

An automated thermal desorption system together with Shimadzu GC-MS-QP 2020 (RTX-5MS capillary column, 60 m × 0.25 mm × 0.25 µm) was used to test the VOCs from the coriander plants. The initial temperature was programmed for 10 min at 50 °C, followed by a temperature increase at the rate of 5 °C min^−1^ to 250 °C, where it was maintained for 5 min. Carrier gas was helium (99.9999%). Electron impact ionization (70 eV) at full scan mode was acquired at 200 °C. The mass scan range was 30–800 amu. Compounds were identified by retention indices with authentic standards or by comparing mass spectra with published data and further identified using the National Institute of Standards and Technology (NIST) database. The relative contents were calculation by comparison with the standard curve of the mixed VOCs standard solution. Mixed VOCs standard solution components included: n-hexane, benzene, trichloroethylene, toluene, octene, ethyl acetate, ethylbenzene, p-xylene, m-xylene, o-xylene, styrene, nonane, isooctanol, undecane, tetradecane, hexadecane (Tanmo Quality Control Standard Material Center, Beijing, China). The identification of the compounds was based on comparison of their retention indices (RI), their retention times (RT) and mass spectra with those obtained from authentic Wiley libraries and the literature [23].

### 2.4. Protocol

**Figure 1:**
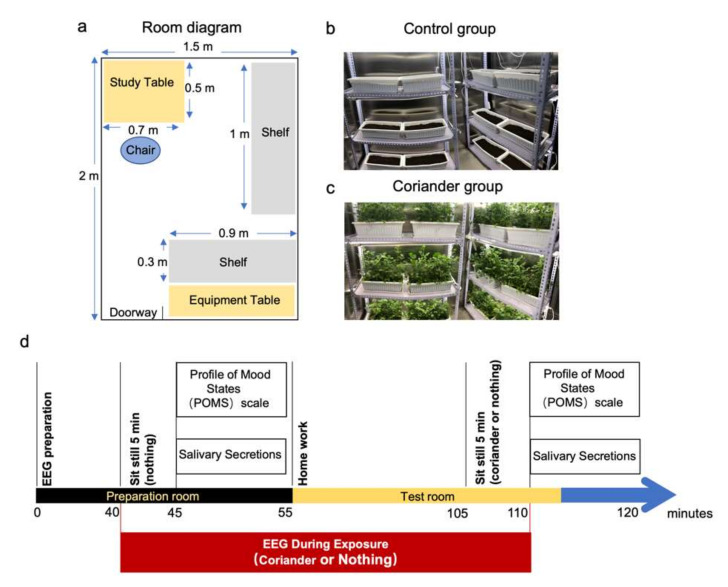
(**a**) The yellow parts indicate the location of the study table and the equipment table. The study table is used for participants to do homework. The equipment table is used to place EEG equipment. The gray parts indicate the position of the coriander shelves (three layers). The blue ellipse shows the position of participants. (**b**) Real scene of the control group. (**c**) Real scene of the coriander group. (**d**) Overview of the protocol design. The blue arrow indicates the total test time. After explaining the test details and protocol, EEG equipment was prepared for 40 min in the preparation room. The participants sat still for 5 min. Subsequently, the participants answered the POMS questionnaire, and the first saliva sample was collected for 10 min. Then the participants entered the test room with or without coriander to do homework for 50 min. After that, the two groups of participants were asked to watch coriander or shelves and sit still for 5 min. Finally, the participants answered the POMS questionnaire and collected the second saliva sample.

The tests were conducted in two rooms (preparation room and test room, length 2 m, width 1.5 m, height 2.5 m) with stainless walls and ceilings of “lunar palace 1”. The layouts and environmental conditions of the two rooms were the same except that were 12 pots of coriander in the coriander group (Figure 1). There were no significant differences in temperature, relative humidity, and CO_2_ levels between the two groups (Table 1). All the participants of the control and coriander groups performed preparation and basic index inspections in the preparation room first and then entered the test room.

The overall experimental protocol, which took 120 min, is shown in Figure 1d. The test starts at 2:00 p.m. The experiment was carried out in five stages: in the first stage, after explaining the details and protocol of the test to each participant, 64-electrode EEG was prepared in the preparation room. The participants sat still for 5 min and subsequently answer the Profiles of Mood States (POMS). At the same time, the first saliva sample was collected. Participants then entered the test room with or without coriander to do homework for 50 min respectively. After that, participants were asked to watch coriander or nothing and sit still for 5 min. Lastly, they answered the POMS questionnaire and collected the second saliva sample. Participants in the coriander group could watch the plants and smell their scents at any time during their homework.

### 2.5. Subjective Evaluation

Depressive and anxiety levels of participants in the preceding week were measured by the Self-Rating Depression Scale (SDS) and the Self-Rating Anxiety Scale (SAS) [24]. The SDS is a quantitative measure of depressive symptoms consisting of 20 items [25]. Participants rate each item regarding how they felt during the preceding week using a 4-point scale that ranges from 1 (a little of the time) to 4 (most of the time). The higher the total score, the greater the depressive symptoms. The SAS is a 20-item self-report assessment designed to measure anxiety levels based on cognitive, autonomic, motor, and central nervous symptoms [26]. Each question is scored on a Likert-type scale of 1 (a little of the time) to 4 (most of the time). The higher the total score, the greater the anxiety symptoms severity.

POMS is a psychological rating scale which was usually used to assess short-term and unique emotional states [27]. A brief version of POMS that comprised 40 items was used in this study, describing seven emotional subscales: tension–anxiety (T-A), anger–hostility (A-H), fatigue (F), dejection (D), confusion (C), vigor (V), and self-esteem (S-E). The 4-point scale rated the items of POMS ranging from “not at all” to “extremely”. The total mood disturbance (TMD) score was defined as TMD = (T-A) + (A-H) + (F) + (D) + (C) − (V) − (S-E).

### 2.6. Measurement of Salivary Alpha-Amylase and Cortisol

According to the diurnal variation of salivary alpha-amylase and cortisol, all tests were scheduled to be performed after 2:00 p.m., when the rate of change slows. Both tests were carried out at the same time on different days. Saliva was collected with Salivettes^®^ collection tubes (Sarstedt, Nümbrecht, Germany), and participants needed to chew it for 3 min. All the saliva samples were stored in a −80 °C refrigerator after pretreatment. Salivary alpha-amylase and cortisol were detected using a human alpha salivary amylase and cortisol ELISA kit (Rigorbio Ltd., Beijing, China).

### 2.7. EEG Date Collection and Analysis

Electroencephalogram (EEG) data were recorded from 64 scalp electrodes using a Brain Vision amplifier system (BrainProducts, Gilching, Germany) and an electrode cap (EasyCap, International 10–20 system) with sintered Ag/AgCl electrodes. The EEG was amplified (band-pass 0.1–100 Hz), digitized at a sampling rate of 1000 Hz, and stored for offline analysis. Electrode impedances were kept below 5 kΩ. Electrodes were referenced to the FCz electrode. Eye blinks and movements were monitored with infra-orbital electrodes.

EEG data were evaluated using BrainVision Analyzer 2.0.2 software (BrainProducts, Gilching, Germany). EEG data were digitally filtered with a 40 Hz Butterworth zero phase lowpass and 0.5 Hz highpass, segmented and baseline corrected. After removing segments with very large artifacts (exceeding ± 500 lV), eye movements and blinks were corrected using independent component analysis (ICA) and baseline-corrected again. Furthermore, a semiautomatic procedure for artifact detection was applied (amplitude criterion ± 50 lV, gradient 20 lV/sample), again controlled by visual inspection [28]. Means were then calculated according to experimental and response conditions, referenced to linked mastoids, and the reconstructed FCz reference was added. After preprocessing, fast Fourier transformation converted the signals from the time domain to the frequency domain, where frequencies were used to separate different rhythmic brain activities. Signals were categorized into five frequency bands: delta (1–4 Hz), theta (5–8 Hz), alpha (9–12 Hz), beta (13–25 Hz) and gamma (26–45 Hz). Additionally, the strength of the brain waves detected by each electrode were normalized by the average value, and a brain map was created using these values in order to obtain the mean value for each electrode.

### 2.8. Salivary Amino Acid Determination

AB SciexExionLC^TM^AD liquid chromatography coupled with an AB SciexQTRAP^®^ 6500+ mass spectrometer (AB Sciex, Framingham, MA, USA) was used to detect the levels of salivary amino acid levels. Quality control (QC) samples were used to determine the state of the instrument before injection and balance the system and evaluate the system’s stability during the experiment. QC was to take an equal amount of per saliva sample and mix it into a quality control samples. Samples were injected into an ACQUITY UPLC BEH Amide column (2.1 × 100 mm, 1.7 μm) using a 9-min linear gradient at a flow rate of 0.3 mL/min. The eluents for the positive polarity mode were eluent A (0.1% formic acid in 5 mM ammonium acetate aqueous solution) and eluent B (0.1% formic acid in acetonitrile). The AB SciexQTRAP^®^ 6500+ mass spectrometer was operated in positive ionization mode with spray voltage of 5.5 kV, capillary temperature of 550 °C, auxiliary gas pressure of 60 psi, atomizing gas pressure of 50 psi, and curtain gas pressure of 35 psi.

### 2.9. Data Analysis

We compared the differences between control and coriander groups using independent *t*-tests in this between-participants design. Paired *t*-tests identified the differences before and after homework in the same group. The saliva index detection levels and subjective emotional evaluation data were calculated as the amount of change before and after treatment. Pearson’s correlation coefficient was performed to evaluate the correlation between the subjective emotional evaluation and the saliva metabolome. *T*-tests and Pearson’s correlation were performed in SPSS 2. Statistical validity was established at *p*-value < 0.05, and for the *t*-test with Cohen’s d, d = 0.2 was a small effect, 0.5 a medium effect, and 0.8 a large effect.

## 3. Results

### 3.1. Main VOCs of Coriander Plants

Coriander plants VOCs was analyzed by GC-MS. Representative gas chromatography–mass spectrometry (GC–MS) total ion chromatograms (Figure S1). From the main of 22 separated peaks, alcohols, terpenoids, and esters were the predominant class of compounds, with 2-ethyl-1-hexanol (15.60%) being the major component (Table 2). 2-ethyl-1-hexanol was often found in the natural VOCs of many plants. Other important substances were: d-limonene (9.58%), eucalyptol (8.97%), benzyl alcohol (6.16%), isophorone (6.06%), dimethyl glutarate (5.03%), α-terpineol (4.45%), styrene (3.97%), methyl methacrylate (3.20%), α-pinene (3.17%). The concentration of VOCs per cm^3^ of coriander plants in the test room was about 0.82 ng. The smell of d-limonene was described as a light lemon smell, α-pinene having a moderate resin scent.

### 3.2. Effects of Coriander Plants on Subjective Emotion

**Figure 2:**
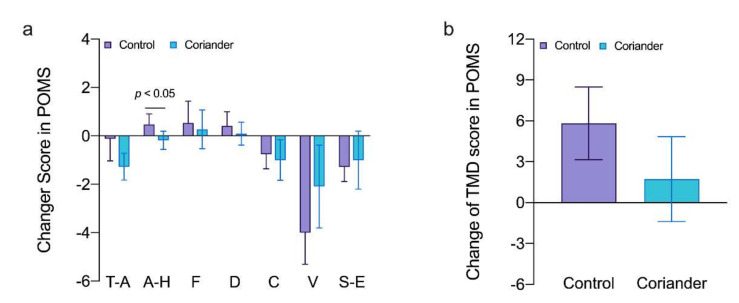
(**a**) Subscale scores for the profile of mood states (POMS) scale in the coriander and control groups (means ± SD). T-A, tension-anxiety; A-H, anger-hostility; F, fatigue; D, dejection; C, confusion; V, vigor; S-E, self-esteem; (**b**) Comparison of the total mood disturbance (TMD) in the profile of mood state (POMS) questionnaire in the two groups. Data are presented as variation ± standard error (*n* = 13). Independent *t*-tests were used to identify the significant differences between the two groups.

More positive psychological relaxation occurred for the stimulus involving coriander plants (Figure 2). Among the subcategories, A-H was lower when performing the task in the coriander group and there was a statistically significant difference (*p* < 0.05, 95% Cl = 0.01; 1.18, Cohen’s d = 1.62, r = 0.63). Regarding other negative emotions and TMD scores, the coriander group tended to be lower than the control group, but there was no statistical difference.

### 3.3. Effects of Coriander Plants on EEG

**Figure 3:**
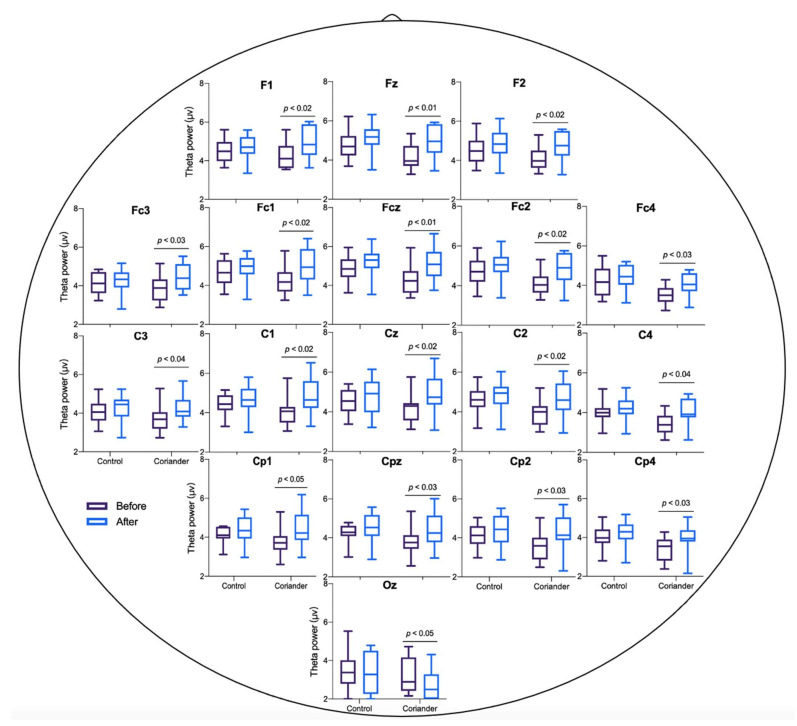
Theta wave power before and after homework in the group of coriander and control. The mean power in theta waves during the sit still for 5 min. Data presented as variation ± standard error (*n* = 10). Paired *t*-tests were used to identify the significant differences before and after in the same groups.

**Figure 4:**
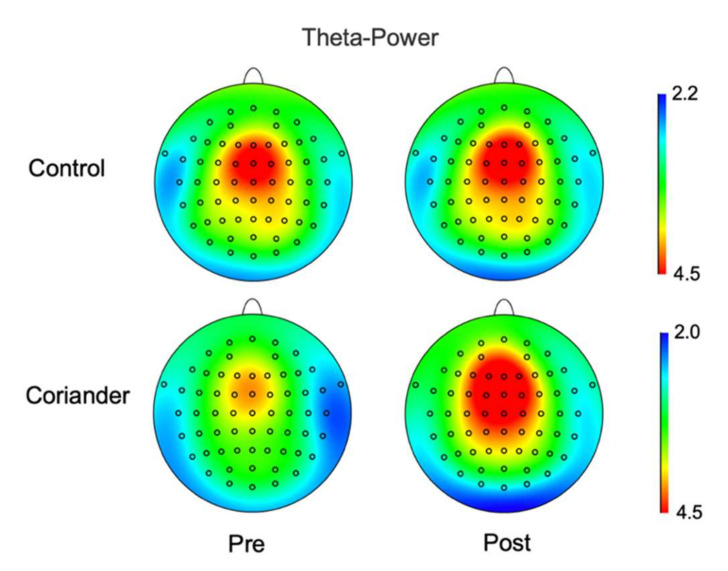
Theta wave power maps before and after homework in the coriander and control groups. The mean power in theta waves during the sit still for 5 min was analyzed and mapped it on the brain. Data presented as a variation (*n* = 10).

The EEG power of theta and alpha was calculated for the control and coriander groups before and after the experiment. Figure 3 and Table S1 show the power of theta brainwaves in two groups, and Figure 4 presents the topographic map. The topographic map showed an obvious change in theta power in the coriander group, particularly in the frontal, frontal central, central, and central parietal areas. The alpha wave power change also showed the same trend as theta, but the difference was not significant (Figure S2).

### 3.4. Effects of Coriander on Salivary Alpha-Amylase and Cortisol

**Figure 5:**
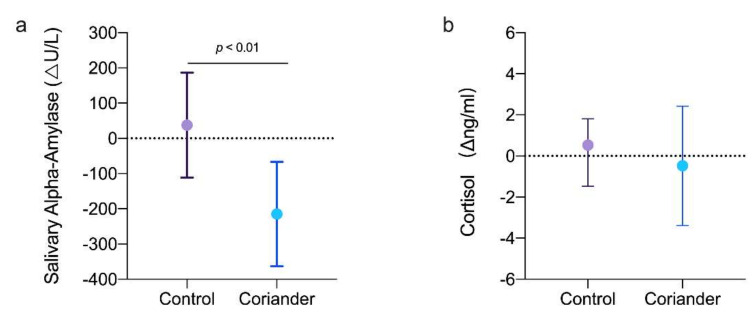
(**a**) Changes in salivary alpha-amylase (Δng/mL) between the coriander and control groups. (**b**) Changes in salivary cortisol (Δng/mL) between the group of coriander and control groups. Data presented as variation ± standard error (*n* = 13). Independent *t*-tests were used to identify the significant differences between the two groups.

Salivary alpha-amylase and cortisol indicated the physiological changes related to emotion. Figure 5 shows the variation in salivary-alpha amylase and cortisol concentrations after the home work in the two groups. Salivary alpha-amylase and cortisol concentrations showed a reducing trend in the coriander groups, especially the salivary alpha-amylase level (*p* < 0.01, 95% Cl = 132.33; 372.82, Cohen’s d = 5.64, r = 0.94), but they showed an increasing trend in the control group.

### 3.5. Effects of Coriander on Salivary Amino Acids

**Figure 6:**
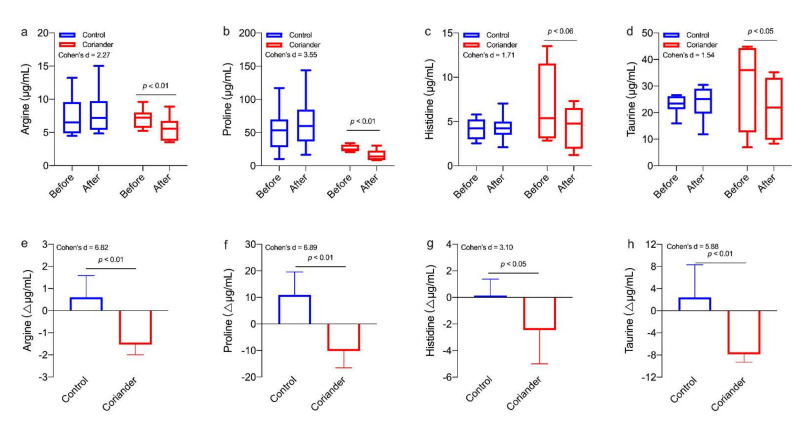
(**a**) Comparison of different salivary amino acids before and after the homework (∆µg/mL). Similar results were shown in (**b**–**d**). Paired *t*-tests were used to identify the significant differences before and after homework in the same group. (**e**) Changes in salivary metabolomics between the coriander and control groups (∆µg/mL). Similar results were shown in (**f**–**h**). Independent *t*-tests were used to identify the significant differences between the two groups. Data presented as variation ± standard error (*n* = 6).

We also analyzed the differences in amino acids of saliva metabolites between the control group and the coriander group (Figure 6). The results showed that the argine (*p* < 0.01, 95% Cl = 1.16; 3.13, Cohen’s d = 6.82, r = 0.95), proline (*p* < 0.01, 95% Cl = 11.37; 30.99, Cohen’s d = 6.89, r = 0.96), histidine (*p* < 0.05, 95% Cl = 0.01; 5.16, Cohen’s d = 3.10, r = 0.84) and taurine (*p* < 0.01, 95% Cl = 4.75; 15.78, Cohen’s d = 5.88, r = 0.95) contents were significantly different between two groups. In addition, the contents of these four amino acids before and after the homework were also significantly different in the coriander group, but not in the control group.

### 3.6. Emotion Correlates with Salivary Amino Acids

**Figure 7:**
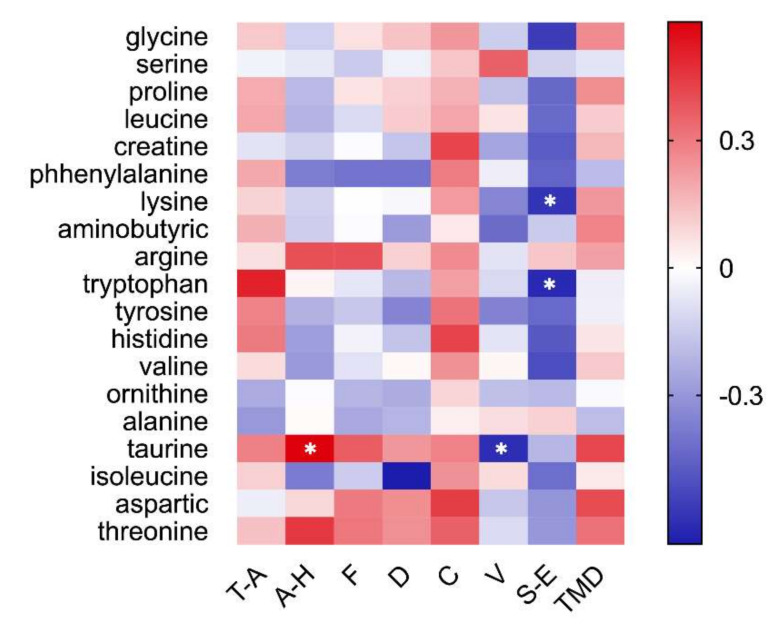
Correlation between subjective emotion and salivary metabolomics. Heat maps exhibit significant statistical correlation values (r). Red squares indicate positive correlation, white squares indicate no correlations, and blue squares indicates negative correlation. The deeper color means a greater correlation (* *p* < 0.05). Pearson’s correlation coefficient and regression analysis were performed to evaluate the connection between subjective emotional evaluation and saliva metabolome.

The results revealed between subjective emotion and salivary metabolomics (Figure 7). The Pearson correlation analysis showed that anger was significantly and positively correlated with taurine (r = 0.58, *p* < 0.05), and vigor was significantly and negatively correlated with taurine (r = −0.61, *p* < 0.05). At the same time, self-esteem was significantly and negatively correlated with tryptophan (r = −0.62, *p* < 0.05) and lysine (r = −0.58, *p* < 0.05).

## 4. Discussion

This study evaluated changes in subjective emotion, brain electrophysiology, and salivary secretion to reveal the intervention effect of coriander plants on people’s emotions and the mechanism underlying the effect. It also analyzed the VOCs of coriander plants and the correlation between emotion changes and salivary secretions.

The results of this study showed that coriander plants could reduce the negative emotions and the theta power in the occipital area. Theta frequency band was related to emotional processing, working memory, and other memory processes [29]. Theta waves appear when people relax after tension, creativity, learning, and internal experiences [30]. Theta and alpha activity increase in the frontal lobes represented focused attention and positive emotions [31]. The increase in theta power caused by coriander plants may be related to their color (Figure 3 and Figure 4). The color of the natural environment, especially green color, was related to emotional improvement. Environmental Psychology points out that a green space environment has a significant restorative impact on mental fatigue and stress [32]. The green color can make people feel relaxed by reducing the impact on the visual system and the activity of neurons of the amygdala and visual cortex [33]. When participants observed a green plant in an indoor space, they experienced an improved emotional state, and their brains were more active in the presence of green than in the presence of white, yellow, pink, or red [30]. Although positive emotions showed a decreasing trend in both groups, this may be caused by working in a claustrophobic environment for one hour continuously (Figure 2a). However, the reduction of positive emotion in the coriander group was lower than in the control group, which can explain the positive regulation of coriander plants on emotion. Hence, coriander plants may reduce negative emotions by affecting brain activity through visual stimuli, although this needs to be confirmed with more evidence.

Moreover, since the olfactory system was closely related to the emotional regions of the brain, fragrance was one of the important factors in emotional regulation [34]. The main component of coriander essential oil was reported to be linalool [35]. It has been suggested that inhaling linalool rich essential oils may counteract anxiety [36]. Linalool was also detected in the volatiles of coriander plants in this experiment, but its low level may be caused by the different extraction methods and parts. Coriander essential oil was commonly obtained from coriander seeds after extraction, while the volatiles naturally released from the coriander plant were tested in this study. Therefore, it was slightly different from the previously reported linalool content [35]. 2-ethyl-1-hexanol might be the main component of the volatile of coriander plants in this study. In previous reports, higher levels of 2-ethyl-1-hexanol were detected also in the volatiles of both fresh tzitzilché flower (14.6%) and tree peony flower (19.76%), which was similar to the results of this study [37,38]. Furthermore, d-limonene and pinene are also commonly used in aromatherapy to relieve anxiety emotion and have some sedative effects [39]. Terpineol and eucalyptol have also been reported to have medicinal value in the treatment of anxiety emotion [40]. The psychological changes stimulated via fragrance inhalation are mainly associated with regulating the olfactory nervous system and subsequent alteration of neuronal activity [41]. Previous scientific studies reported that fragrance from various plant species such as Lavandula officinalis significantly increase theta wave activity and decrease stress scores [42]. Plant volatiles are currently mostly used to manage chronic pain, depression, cognitive disorders, anxiety, insomnia and stress-related disorders [36]. Therefore, the volatiles of coriander plants may also be an important factor in regulating mood. However, the regulation mechanism of coriander volatile on emotion and which component plays a key role need to be further studied.

The limbic system activates the hypothalamus, which controls the stress response systems, the sympathetic adrenal medullary (SAM) response system, and the hypothalamic pituitary adrenal system (HPA) [43]. Free cortisol in saliva reflects HPA activity, while salivary alpha-amylase reflects SAM activity [44]. In response to stress, the body activates the HPA of the sympathetic nervous system and releases stress hormones (control). At the same time that HPA is reacting, autonomic nervous system activation also stimulates the adrenal gland leading to a high salivary alpha-amylase activity [45]. In this study, coriander plants reduced salivary alpha-amylase and cortisol levels (Figure 5), thereby reducing stress (Figure 2a). This finding was consistent with previously reported results that stress positively correlates with salivary alpha-amylase and cortisol levels [12,41]. Green color may be one of the main reasons that coriander plants reduce salivary alpha-amylase and cortisol levels. Research have shown that the green color of plants could restore mental state and relieve work pressure [46]. Humans receive information from the environment mainly through visual perception. The green color of coriander plants can be sensed by ipRGCs and projected to the paraventricular nucleus of the hypothalamus, which may downregulate salivary alpha-amylase and cortisol secretion.

There is a long history and clear evidence of altered endocrine factors (e.g., HPA) and metabolic dysregulation in mood disorders [47]. Early studies using metabolomics have identified the panel of metabolites associated with depression-like behavior in animal models [48,49] and the metabolic in major depressive disorder patients [50]. Depressed individuals had higher taurine, proline, glycine, and alanine levels [51,52]. While the HPA of depressed rats was over-activated, the urine metabolome also changed significantly, including the arginine and proline metabolic pathways [53]. Our results were similar to those previously reported on the metabolism of depression. The results showed that the arginine, proline, histidine, and taurine content were significantly lower in the coriander group than the control group (Figure 6). In addition, the content changes of these four amino acids before and after the homework also showed significant differences between the coriander and control groups. It showed that coriander plants could regulate the level of metabolites related to emotion. Further correlation analyses showed that anger significantly and positively correlated with taurine, and vigor significantly and negatively correlated with taurine (Figure 7), suggesting that emotional changes may affect the metabolism of salivary amino acids. This finding was consistent with other reported results [50,51,52].

## 5. Conclusions

This study showed that coriander plant could influence the activity of brain electrophysiological and salivary secretion through its VOCs to improve people’s negative emotions. Particularly, coriander plants can reduce anger and taurine in saliva, and increase theta wave power in the frontal, frontal central, central, and central parietal areas. However, our study had some limitations. The sample size was not large enough, and the effective concentration of VOCs in coriander plants was not determined. Future research should recruit more participants and depressed patients to further analyze the separate regulation mechanism on emotions of coriander plants through vision and the olfactory pathway. Meanwhile, the direct relationship between the VOCs concentration of coriander plants and the effect of emotion regulation should be analyzed.

## Figures and Tables

**Table 1:** Baseline measurements of participants (*n* = 26) and environment quality of the experiment.

Parameters	Control Group		Coriander Group		*p*-Value
	Mean	SD	Mean	SD	
Number	13	-	13	-	-
Female	7	-	7	-	-
Male	6	-	6	-	-
Age (years)	20.16	0.42	22.72	1.05	0.27
Height (cm)	169.75	4.71	171.45	3.01	0.60
Body weight (kg)	62.23	4.68	60.18	4.31	0.56
BMI (kg/m^2^)	21.58	1.12	20.18	0.98	0.22
SAS	38.53	2.02	38.50	2.96	0.98
SDS	41.46	2.09	40.92	1.96	0.73
Temperature (°C)	20.22	0.62	20.13	0.56	0.71
Relative humidity (%)	50.77	3.83	52.45	2.09	0.13
CO_2_ concentration (ppm)	769.44	31.13	737.66	38.96	0.10

SDS: Self-Rating Depression Scale; SAS: Self-Rating Anxiety Scale.

**Table 2:** Main volatile components of coriander plants.

Compounds	Chemical Formula	RT (min)	RI	RC (%)	CAS
Longifolene	C_15_H_24_	36.46	1417.58	2.20	475-20-7
1,3,5-Benzetriol, 3TMS derivative	C_15_H_30_O_3_Si_3_	32.49	1265.55	1.66	10586-12-6
2-Ethylhexyl acrylate	C_11_H_20_O_2_	30.82	1146.97	1.59	103-11-7
α-Terpineol	C_10_H_18_O	29.88	1136.51	4.45	98-55-5
Dimethyl glutarate	C_7_H_12_O_4_	27.86	1114.01	5.03	1119-40-0
Lsophorone	C_9_H_14_O	27.59	1110.99	6.06	78-59-1
1,2,3,5-Tetramethylbenzene	C_10_H_14_	27.37	1108.63	2.82	527-53-7
3-Hydroxymandelic acid, 3TMS derivative	C_17_H_32_O_4_Si_3_	27.22	1106.86	2.69	68595-69-7
Linalool	C_10_H_18_O	26.66	1100.66	0.60	78-70-6
Acetophenone	C_8_H_8_O	25.58	987.55	1.95	98-86-2
γ-Terpinene	C_10_H_16_	25.27	983.73	1.10	99-85-4
Benzyl alcohol	C_7_H_8_O	24.36	972.51	6.16	100-51-6
Eucalyptol	C_10_H_18_O	24.30	971.76	8.87	470-82-6
D-Limonene	C_10_H_16_	24.16	870.37	9.58	5989-27-5
2-Ethyl-1-hexanol	C_8_H_18_O	24.07	968.90	15.60	104-76-7
α-Pinene	C10H16	20.17	930.96	3.17	80-56-8
Styrene	C_8_H_8_	18.11	805.88	3.92	100-42-5
Furfural	C_5_H_4_O_2_	15.14	639.22	0.75	98-01-1
Methyl methacrylate	C_5_H_8_O_2_	9.18	613.16	3.20	80-62-6
1-Butanol	C_4_H_10_O	7.59	692.01	1.45	71-36-3
1,2-Ethanediol, diformate	C_4_H_6_O_4_	6.35	598.03	6.18	629-15-2
Ethyl Acetate	C_4_H_8_O_2_	6.61	618.02	2.54	141-78-6
Other volatile components	-	-		t	-

RT = retention times; RI = retention indices; RC = relative concentration; t—trace (<0.06%).

## Data Availability

Data is contained within the article or Supplementary Materials.

## Supplementary Materials

The following are available online at https://www.mdpi.com/article/10.3390/biology10121283/s1, Table S1: The Theta power before and after homework in the group of coriander and control, Figure S1: Representative gas chromatography–mass spectrometry (GC–MS) total ion chromatograms, Figure S2: Alpha power maps before and after homework in the coriander and control groups.
