# Supplementary material for: Effect of Coriander Plants on Human Emotions, Brain Electrophysiology, and Salivary Secretion
Source: Biology (Basel). 2021 Dec 6;10(12):1283. doi: 10.3390/biology10121283 (PMC8698652; doi:10.3390/biology10121283)
Supplement: Supplementary file 1 [file biology-10-01283-s001.zip › biology-1471899-supplementary.pdf]

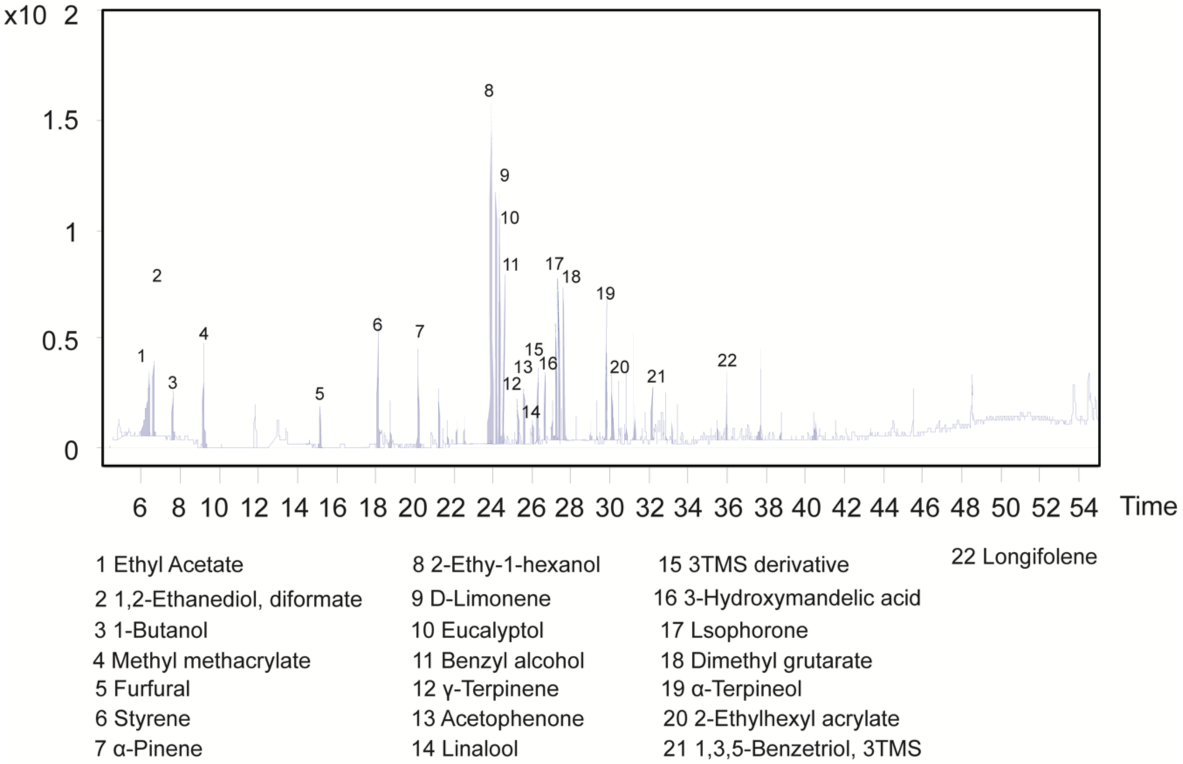


Figure. S1 Representative gas chromatography–mass spectrometry (GC–MS) total ion chromatograms.

Table S1 The Theta power before and after homework in the group of coriander and control. Data presented as variation ± standard error (N = 10).

| **Electrode** | **Control group** | | | | **Coriander group** | | | |
|---|---|---|---|---|---|---|---|---|
| | Before Theta power (μV) | After Theta power (μV) | P Value | 95% CI | Before Theta power (μV) | After Theta power (μV) | P Value | 95% CI |
| **F1** | 4.48(0.24) | 4.70(0.25) | 0.32 | -0.70; 0.26 | 4.24(0.23) | 4.98(0.27) | 0.02 | -1.32; -0.15 |
| **F2** | 4.51(0.27) | 4.83(0.31) | 0.17 | -0.87; 0.17 | 4.09(0.19) | 4.74(0.23) | 0.02 | -1.18; -0.11 |
| **F3** | 4.03(0.24) | 4.11(0.21) | 0.67 | -0.51; 0.34 | 3.86(0.20) | 4.45(0.23) | 0.04 | -1.14; -0.05 |
| **Fz** | 4.73(0.28) | 5.11(0.31) | 0.09 | -0.83; 0.08 | 4.18(0.21) | 4.98(0.26) | 0.01 | -1.36; -0.25 |
| **FC1** | 4.65(0.26) | 4.89(0.29) | 0.18 | -0.62; 0.14 | 4.23(0.23) | 5.01(0.29) | 0.02 | -1.37; -0.16 |
| **FC2** | 4.70(0.29) | 4.98(0.33) | 0.21 | -0.75; 0.19 | 4.09(0.19) | 4.83(0.26) | 0.02 | -1.29; -0.17 |
| **FC3** | 4.11(0.23) | 4.25(0.26) | 0.39 | -0.50; 0.22 | 3.86(0.21) | 4.4m6(0.24) | 0.03 | -1.13; -0.06 |
| **FC4** | 4.19(0.32) | 4.41(0.29) | 0.33 | -0.72; 0.28 | 3.51(0.14) | 4.08(0.19) | 0.03 | -1.09; -0.05 |
| **FCz** | 4.84(0.26) | 5.20(0.33) | 0.08 | -0.77; 0.06 | 4.29(0.24) | 5.13(0.27) | 0.01 | -1.43; -0.22 |
| **C1** | 4.43(0.22) | 4.64(0.32) | 0.26 | -0.62; 0.19 | 4.04(0.24) | 4.81(0.29) | 0.02 | -1.37; -0.17 |
| **C2** | 4.59(0.28) | 4.78(0.34) | 0.35 | -0.66; 0.26 | 3.93(0.20) | 4.62(0.28) | 0.02 | -1.26; -0.12 |
| **C3** | 4.05(0.26) | 4.25(0.29) | 0.28 | -0.61; 0.20 | 3.69(0.23) | 4.27(0.23) | 0.04 | -1.11; -0.03 |
| **C4** | 4.00(0.23) | 4.18(0.28) | 0.27 | -0.53; 0.17 | 3.43(0.18) | 4.03(0.21) | 0.04 | -1.15; -0.05 |
| **Cz** | 4.54(0.25) | 4.83(0.36) | 0.11 | -0.65; 0.08 | 4.14(0.24) | 4.90(0.32) | 0.02 | -1.36; -0.14 |
| **CP1** | 4.07(0.18) | 4.33(0.29) | 0.17 | -0.66; 0.14 | 3.76(0.23) | 4.43(0.29) | 0.05 | -1.34; -0.01 |
| **CP2** | 4.11(0.24) | 4.32(0.34) | 0.07 | -0.64; 0.02 | 3.60(0.23) | 4.29(0.29) | 0.03 | -1.28; -0.09 |
| **CP4** | 4.01(0.26) | 4.19(0.30) | 0.25 | -0.55; 0.17 | 3.36(0.19) | 3.94(0.23) | 0.03 | -1.08; -0.05 |
| **CPz** | 4.22(0.21) | 4.41(0.33) | 0.09 | -0.65; 0.06 | 3.80(0.23) | 4.45(0.28) | 0.03 | -1.23; -0.05 |
| **P2** | 4.05(0.28) | 4.26(0.34) | 0.19 | -0.56; 0.13 | 3.44(0.21) | 3.98(0.22) | 0.04 | -1.04; -0.03 |
| **Oz** | 3.37(0.46) | 3.27(0.44) | 0.71 | -0.49; 0.69 | 3.16(0.28) | 2.60(0.38) | 0.05 | -0.01; 1.14 |

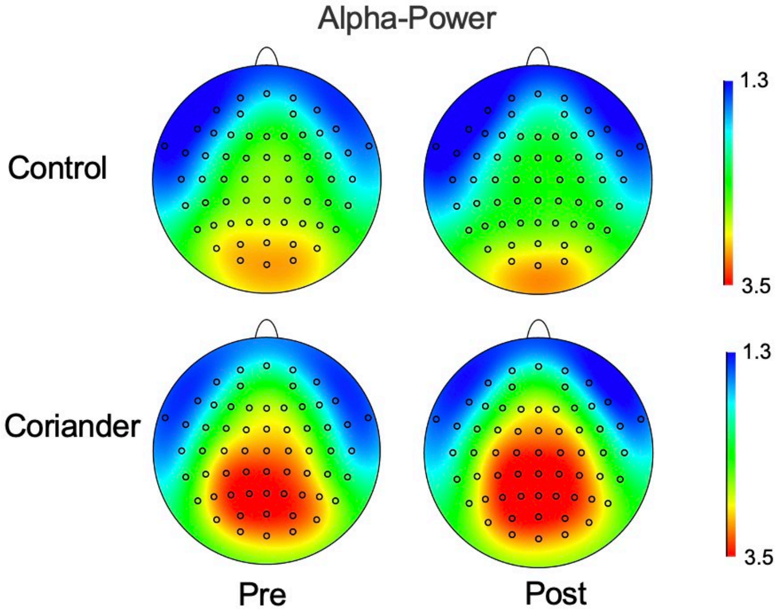


Figure. S2 Alpha power maps before and after homework in the coriander and control groups. The mean power in alpha during the sit still for 5 min was analyzed and mapped on the brain. Data was presented as variation (N = 10).
